# The potential presence of infection may be indicated through non-invasive prediction of procalcitonin and C-reactive protein levels within the initial three days after cervical cerclage: a retrospective case-control study

**DOI:** 10.1186/s12884-024-06668-9

**Published:** 2024-07-11

**Authors:** Xiucong Fan, Yabin Ma, Yunxia Zhu, Weijun Tang, Xiaohui Dong, Ming Liu

**Affiliations:** 1 Department of Pharmacy, Shanghai East Hospital, Tongji University School of Medicine, Shanghai, 200123 China; 2 Department of Obstetrics, Shanghai East Hospital, Tongji University School of Medicine, Shanghai, 200123 China; 3 Department of Laboratory Medicine, Shanghai East Hospital, Tongji University School of Medicine, Shanghai, 200123 China

**Keywords:** Cervical insufficiency, Post-cervical cerclage infections, Infection indicators, Propensity score matching, Receiver operating characteristic

## Abstract

**Purpose:**

To identify which non-invasive infection indicators could better predict post-cervical cerclage (CC) infections, and on which days after CC infection indicators should be closely monitored.

**Methods:**

The retrospective, single-center study included 619 single-pregnancy patients from January 2021 to December 2022. Patients were categorized into infected and uninfected groups based on physicians’ judgments of post-CC infections. Registered information included patient characteristics, cervical insufficiency history, gestational age at CC, surgical method (McDonald/Shirodkar), purpose of CC, mid-pregnancy miscarriage/preterm birth, infection history or risk factors, and infection indices on days 1, 3, 5, and 7 after CC. Propensity score matching (PSM) was applied to reduce patient characteristic bias. Statistical analysis of C-reactive protein (CRP), white blood cell (WBC), neutrophil count (NEU), percentage of neutrophil count (NEU_P), interleukin-6 (IL-6), and procalcitonin (PCT) in the infected group compared with the uninfected group was performed using chi-square tests and t-tests. Receiver operating characteristic (ROC) curves were used to further assess the diagnostic value of CRP, PCT, and CRP-PCT in combination.

**Results:**

Among the 619 included patients, 206 patients were matched using PSM and subsequently assessed. PCT values on day 1 and day 3 after CC exhibited significant differences between the two groups in two statistical ways (*P* < 0.01, *P* < 0.05). The CRP levels on day 1 were significantly higher in the infected group compared to the uninfected group in two statistical ways (*P* < 0.05). On day 3, the mean CRP value was significantly elevated in the infected group compared to the uninfected group (*P* < 0.05). Analyses of IL-6, WBC, NEU, and NEU_P did not yield clinically significant results. The area under the ROC curves for CRP, PCT, and CRP-PCT on day 1 and day 3 were all below 0.7. In the preventive CC group, the AUC values of CRP and CRP-PCT obtained on d1 were found to be higher than 0.7, indicating moderate diagnostic accuracy.

**Conclusion:**

For women after CC surgery, especially of preventive aim, increased serum CRP and PCT levels from post-CC day 1 to day 3 may signal a potential postoperative infection, warranting close monitoring.

## Background

Cervical insufficiency (CI) refers to the inability of the cervix to maintain pregnancies until full term due to anatomical or functional abnormalities [1]. This condition affects approximately 1% of pregnant women [2] and is responsible for approximately 10–25% of spontaneous pregnancy losses in the second trimester and premature births in the absence of uterine contractions. Typical clinical manifestations include cervical dilation and the protrusion of the amniotic sac into the vagina [1, 3]. Despite extensive research, there is still no consensus on the specific mechanisms underlying CI. Current understanding suggests that acquired causes of CI include a history of cervical surgery, mechanical dilation during induced abortion or hysteroscopy, and cervical lacerations during delivery. Congenital causes primarily involve congenital Mullerian tube hypoplasia, cervical collagen and elastin deficiencies, and exposure to diethylstilbestrol in utero [4].

CI has a significant impact on the physical and psychological health of pregnant women. Previous research has reported that 13.1% of pregnant women in low- and middle-income countries experience depression [5], with a prevalence of antenatal depression in both parents at 1.72% [6]. Since the Chinese government implemented the two-child policy in 2016, the incidence of mid-pregnancy miscarriages due to CI and premature births has increased. It is reasonable to assume that families affected by CI experience greater psychological and financial pressures due to the fear of pregnancy loss and the high cost of caring for premature infants. Consequently, obstetricians face substantial challenges in preventing pregnancy loss in women previously diagnosed with CI and treating CI in those who develop it.

Currently, CI management methods are categorized as surgical and non-surgical. Non-surgical approaches typically involve activity restriction and bed rest, but research has shown their ineffectiveness and discouraged their use [7]. Cervical pessaries have also been used as a non-surgical option, but limited and conflicting data have questioned their benefits [8]. Cervical cerclage (CC) is a common surgical method for CI, with McDonald’s and Shirodkar’s procedures being the most frequently employed. These procedures offer advantages such as minimal trauma, rapid recovery, simplicity, and the ability to perform them during pregnancy. Effective CC is crucial in extending pregnancy duration, reducing premature birth rates, and decreasing neonatal complications [1]. Consequently, surgical management is more widely favored for CI compared to non-surgical methods.

Successful post-surgical management depends on several factors, with infection management playing a pivotal role. Local infections at the surgical site increase the risk of chorioamnionitis, a significant complication in CI management [9]. C-reactive protein (CRP) is a predictive marker for infection and is associated with an increased incidence of chorioamnionitis [10]. Kobayashi M et al. [11] found that serum CRP levels on post-cerclage day 1 and day 2 correlated with subsequent very preterm births. Given the importance of postoperative infection monitoring, obstetricians often conduct frequent monitoring of serum infection markers, including CRP and other indicators.

To address the issue of excessive monitoring, we conducted a retrospective analysis of post-CC surgical infection indicators to determine which serum infection marker could better predict postoperative infections and on which post-surgery days these markers should be monitored. This approach aims to save more medical resources and enhance the patient’s overall medical experience.

## Methods

We adhered to the STROBE Statement for cohort studies in conducting this study.

### Study design and setting

This retrospective cohort study was conducted at Tongji University Affiliated East Hospital, a tertiary medical centre in Shanghai. This centre is currently one of the largest premature delivery centers in China and is renowned for diagnosing and treating CI. The study design received approval from the Ethics Committee of Tongji University Affiliated East Hospital, with approval number 2023-072, and was exempted from informed consent.

### Subjects and data collection

The research subjects included in-hospital patients admitted to the Obstetrical Department for CC between January, 2021 and December, 2022. These patients were divided into two cohorts: the infected group and the uninfected group. The classification into the infected or uninfected group depended on consultation recommendations from the antibiotic use group. Specifically, patients suspected of intrauterine infection (IAI) and surgical site infection (SSI) were categorized as infected. Criteria for suspected IAI included maternal temperature ≥ 39.0 °C, or 38.0–38.9 °C with additional risk factors (maternal tachycardia ≥ 100 beats/min or fetal tachycardia ≥ 160 beats/min or maternal white blood cell ≥ 15,000/mm) [12]. Diagnoses excluded the influence of drugs and other infectious diseases. SSI was determined if at least one factor appeared [13]: pus from the surgical site, positive microbial culture from surgical site, discovered through direct exploration, histopathological examination or imaging examination, along with physician judgment on SSI. Exclusion criteria included: (1) patients with twin pregnancies; (2) patients who refused to monitor infection indices after surgery according to physician’s requirements; (3) patients with incomplete medical or surgical records; (4) patients who underwent abdominal cerclage.

We reviewed the medical records of the subjects in the Electronic Medical Record System and handwritten records collected manually. The extracted information included patient identity, age, BMI, residential details, CI history, gestational age for CC, surgical method (McDonald/Shirodkar), purpose of CC, mid-pregnancy miscarriage/preterm birth, infection history, risk factors for infection, and infection indices on day 1, day 3, day 5, and day 7 after surgery. Senior physicians crosschecked the data to prevent information collection bias.

### Cervical cerclage management

In this study, CC indications were evaluated comprehensively based on guidelines [14–16]. Preventive CC, indicated by medical history, is recommended for: (1) patients with a history of 3 or more mid-term miscarriages or premature births; (2) patients with a history of mid-pregnancy miscarriage due to one or multiple painless cervical dilations, excluding factors like labour and placental abruption; (3) patients whose previous indication for CC was painless cervical dilation in the second trimester of pregnancy. Therapeutic CC, indicated by ultrasound, is recommended for patients with a history of spontaneous premature birth before 34 weeks of pregnancy and cervical length < 25 mm before 24 weeks during this pregnancy. Emergency CC, indicated by physical examination, is recommended for patients with painless cervical dilation in mid-pregnancy. Patients and their families should sign informed consent forms before the surgery. Before CC, patients should undergo blood routine tests, vaginal discharge, and urine culture examinations. Preventive and therapeutic CC could be implemented until the results showed negative or the infection was cured. Patients requiring emergency CC do not need test results.

**Fig. 1:**
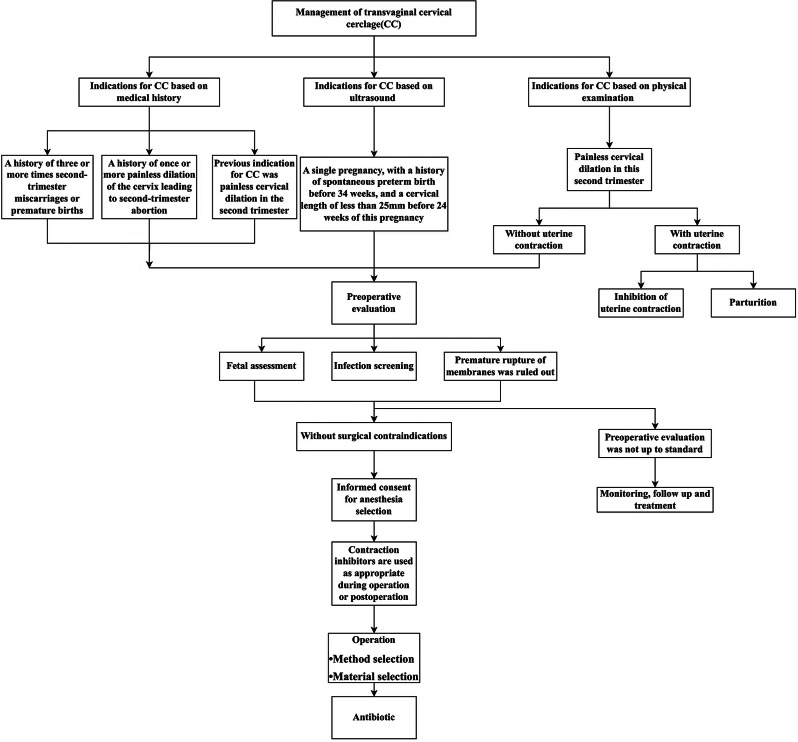
Management process of transvaginal CC

Once CC is decided, the McDonald or Shirodkar method should be chosen based on the patient’s condition. For CC surgery based on ultrasound and physical examination indications, indometacin suppositories could be utilised for patients during and after surgery, especially when uterine contractions occur. Pregnant women who have already received progesterone supplementation treatment before CC will continue to receive progesterone after CC. Cefoxitin is used to prevent infection of the surgical site from before surgery to 48 h post-surgery. Infection indices including C-reactive protein (CRP), white blood cell (WBC), neutrophil count (NEU), percentage of neutrophil count (NEU_P), interleukin-6 (IL-6), and procalcitonin (PCT) are tested on day 1, day 3, day 5, and day 7. When infection occurs, preventive cefoxitin is replaced by therapeutic antibiotics until infection indices return to normal and infection symptoms disappear. If infection control fails, the CC line should be removed (see Fig. 1).

### Specimen testing and instruments

PCT and IL-6 levels were determined using electrochemiluminescence immunometric assay, employing the Roche E801 autoimmune analyser (Roche Diagnostics, Japan). Serum CRP levels were assessed using immunoturbidimetry assay, with the BC-7500(NR) CS analyser (Mindray, China). Serum WBC, NEU, and NEU-P levels were measured using flow cytometry, and the BC-7500(NR) CS analyser (Mindray, China).

### Variables

Receiver operating characteristic (ROC) curves were used to estimate the sensitivity and specificity of the infection indices. The optimal threshold for ROC curves was defined as the value that maximized the sum of sensitivity and specificity. An area under the curve (AUC) value of 1 indicates a perfect test, while a value greater than 0.9 signifies high accuracy, and a value between 0.7 and 0.9 indicates moderate accuracy. Values less than 0.7 indicate low accuracy. The normal ranges for CRP, WBC, NEU, NEU_P, PCT, and IL-6 were defined as 0–10 mg/L, 3.5–9.5 × 10^9/L, 1.8–6.3 × 10^9/L, 40–75%, 0-0.05 ng/ml, and 0–7 pg/ml, respectively.

### Statistics

A total of 619 pregnant patients meeting the criteria were included in this study. We utilized SPSS 25.0 software (SPSS Inc., Chicago, IL, USA) for Windows to perform the propensity score matching (PSM) approach, which aimed to reduce confounding bias between the groups. Logistic regression was used to estimate the propensity score, incorporating age, women with a CI history, gestational age for CC, surgical method, purpose of CC, mid-pregnancy miscarriage, women with a BMI > 28, and women with a history of infection or high-risk factors for infection as variables. We specified 1:1 nearest neighbor matching, halting when the variable fell within the range of 0.0 ± 0.02. We employed the Kolmogorov‒Smirnov test to confirm data normal distribution. If the data followed a normal distribution, they were presented as means and standard deviations (SDs) and analyzed using the t-test. For continuous data that were not normally distributed, they were presented as medians with interquartile ranges (IQRs) and compared using the Mann‒Whitney U test. The chi-square test was used to analyze frequency, rate, or constituent ratio. ROC curves were generated to assess the sensitivity and specificity of CRP and PCT, either individually or in combination, after 1, 3, 5, and 7 days of CC. The ROC curve for combined diagnosis was constructed based on the predicted probability from binary logistic regression analysis of CRP and PCT. P-values less than 0.05 were considered statistically significant.

## Results

**Fig. 2:**
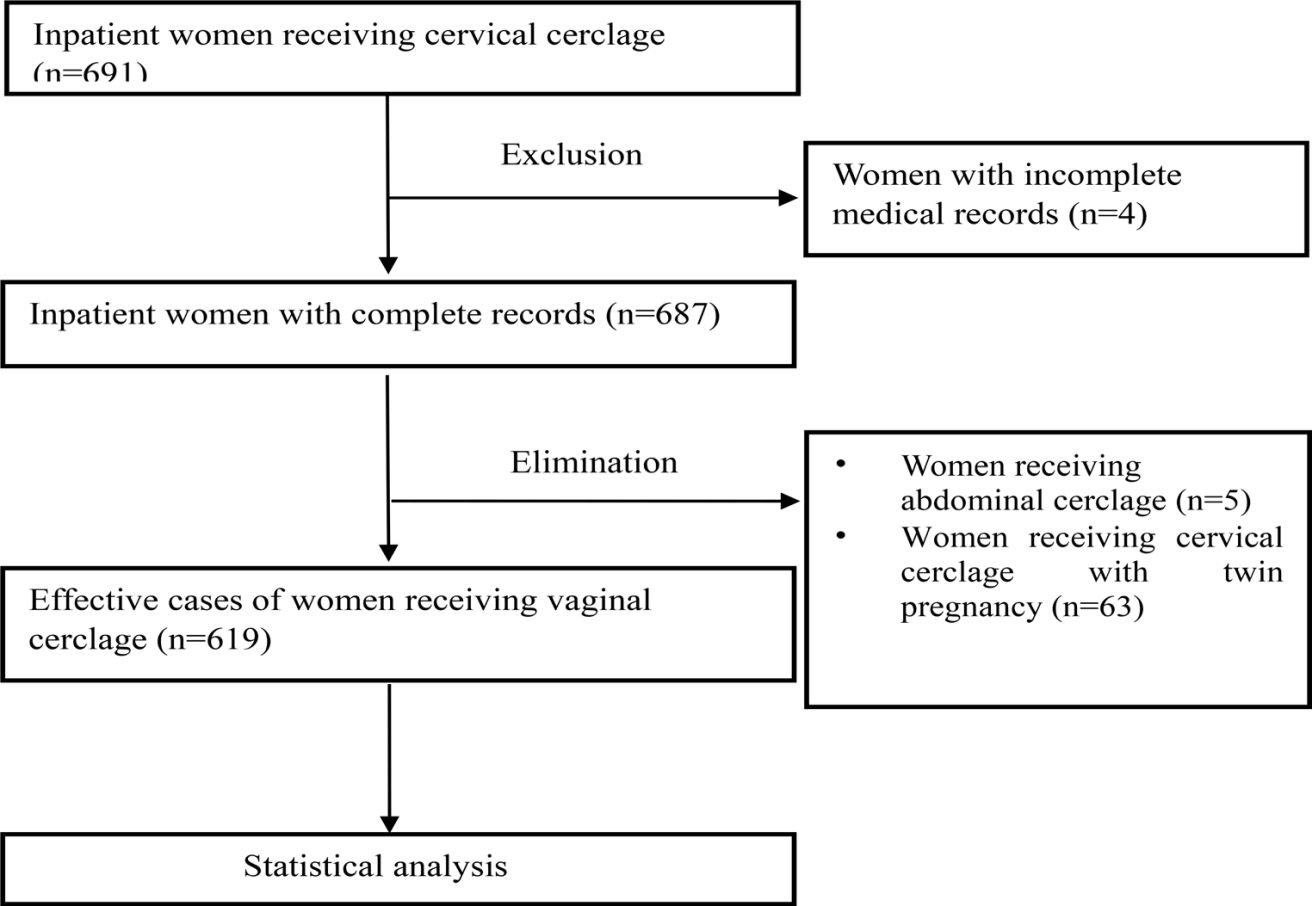
Flow chart of selection of women in the study

**Fig. 3:**
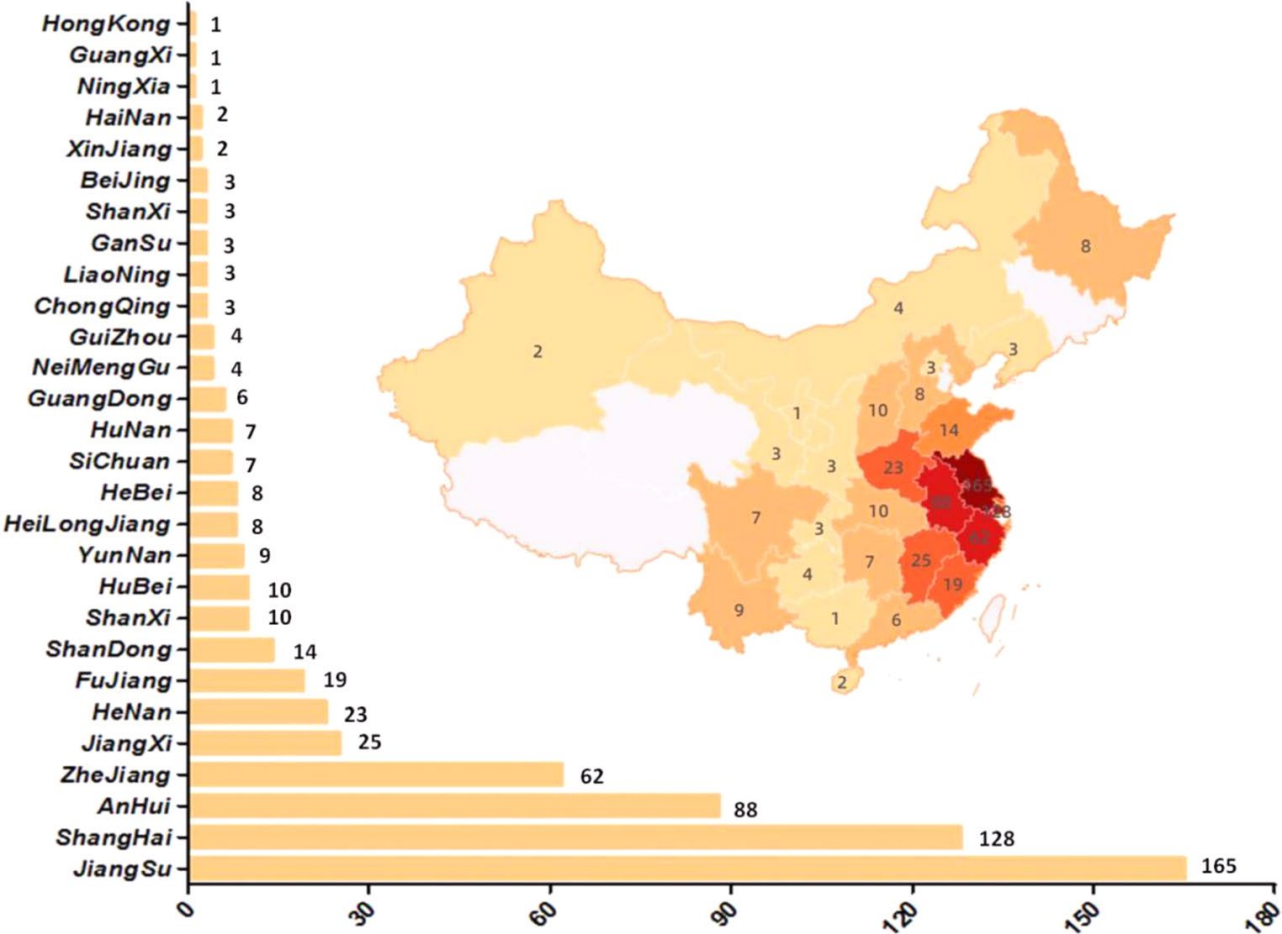
Distribution of included patients (*n* = 619)

In this study, a total of 691 patients were screened, with 619 patients meeting the inclusion criteria and being ultimately included (Fig. 2). As illustrated in Fig. 3, these patients hailed from 28 provinces, cities, and regions in China. The highest proportion of patients (165/619) originated from Jiangsu Province, with Shanghai ranking second (128/619).

**Table 1:** Baseline characteristics before and after PSM in the infected and uninfected groups

Characteristics		Before PSM				After PSM		
		Infected group (n, %)	Uninfected group (n, %)	*P* value		Infected group (n, %)	Uninfected group (n, %)	*P* value
		*n* = 107	*n* = 512			*n* = 103	*n* = 103	
Age (years)		31.7 ± 3.28	31.82 ± 3.89	0.764		31.69 ± 3.46	31.24 ± 4.16	0.4
Women with cervical insufficiency history		83(77.57)	345(67.38)	0.038		79(76.70)	78(75.73)	0.87
Gestation age of cervical cerclage ≤ 16w		30(28.04)	242(47.26)	< 0.001		29(28.16)	23(22.33)	0.336
Surgical method	Macdonald	44(41.12)	68(13.28)	< 0.001		40(38.83)	38(36.89)	0.774
	Shirlock	63(58.88)	444(86.72)			63(61.27)	65(63.11)	
Aim of cervical cerclage	Preventive	30(28.04)	324(63.28)	< 0.001		30(29.13)	32(31.07)	0.761
	Therapeutic	62(57.94)	181(35.35)	< 0.001		62(64.08)	64(62.14)	0.774
	Emergency	15(14.02)	7(1.38)	< 0.001		7(6.79)	7(6.79)	1
Miscarriage preterm birth in mid pregnancy	0	33(30.84)	121(23.63)	0.117		33(32.09)	31(30.10)	0.763
	1	51(47.66)	277(54.10)	0.225		49(47.57)	51(49.51)	0.78
	2	19(17.76)	98(19.14)	0.74		17(16.50)	15(14.56)	0.7
	≥ 3	4(3.74)	16(3.13)	0.744		4(3.84)	6(5.83)	0.517
Women of BMI > 28		21(19.63)	42(8.20)	< 0.001		17(16.50)	16(15.53)	0.849
Women with infection history or high risk factors for infection		33(30.84)	160(31.25)	0.934		31(30.10)	31(30.10)	1

Table 1 presents the characteristics of the patients included in the study. As there were significant differences in the characteristics of women with a history of cervical insufficiency, gestational age of cervical cerclage ≤ 16 weeks, surgical method, purpose of cervical cerclage, and women with BMI > 28 between the two groups (*P* < 0.05), we applied the propensity score matching (PSM) to reduce bias and ensure comparability. Following PSM analysis, no significant differences were observed in the demographic variables between the two groups.

**Fig. 4:**
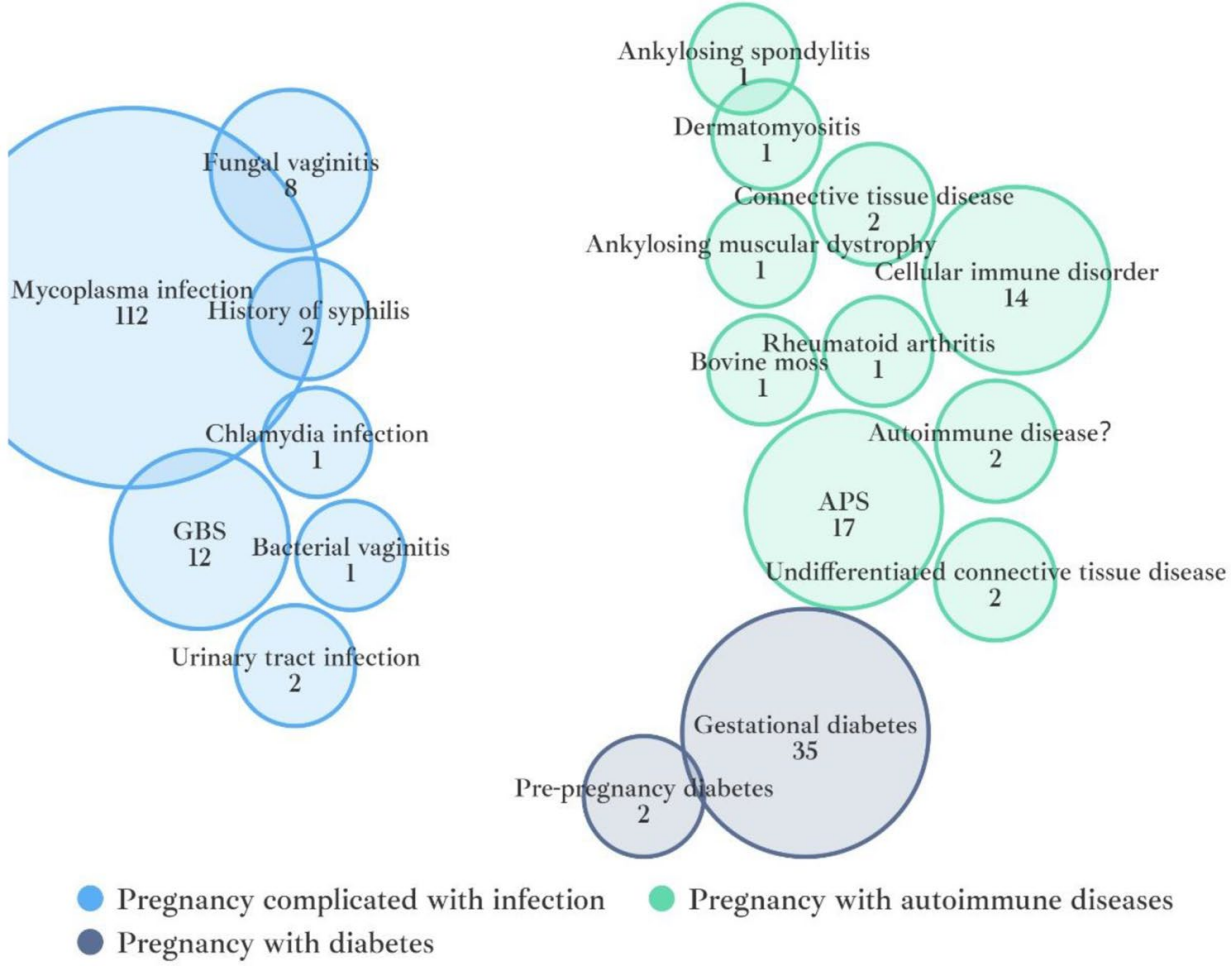
Risk factors for postoperative infection included in the study (*n* = 193). GBS: Group B Streptococcus APS: Antiphospholipid Syndrome

Regarding the composition of the risk factors for postoperative infection shown in Fig. 4, a total of 193 risk factors were considered in the study. Pregnancy complicated with infection represented the highest proportion, followed by pregnancy complicated with autoimmune disease and pregnancy complicated with diabetes. Specifically, mycoplasma infection (112/193), gestational diabetes (35/193), and Antiphospholipid Syndrome (APS) (17/193) ranked as the top three risk factors.

**Table 2:** Abnormal infection indices of the cervical cerclage patients in the infected group and the uninfected group

	d1 after cervical cerclage	d3 after cervical cerclage	d5 after cervical cerclage	d7 after cervical cerclage
**CRP(0-10 mg/L)**	*n* = 191	*n* = 164	*n* = 92	*n* = 59
Uninfected group	38/97(39.18%)	42/79(56.96%)	11/39(28.21%)	5/23(21.74%)
	10.36 ± 0.72	12.11 ± 0.79	9.59 ± 1.66	6.49 ± 1.38
Infected group	52/94(55.32%)	53/85(62.35%)	23/53(43.40%)	14/36(38.89%)
	14.76 ± 0.56	18.92 ± 2.64	15.61 ± 2.88	12.87 ± 2.50
*χ2 value*	*4.366*	*0.296*	*1.621*	*1.187*
*P value*	*<0.05*	*0.586*	*0.203*	*0.276*
WBC(3.5–9.5*109/L)	*n* = 191	*n* = 164	*n* = 92	*n* = 59
Uninfected group	61/97(62.89%)	29/79(36.71%)	17/39(43.59%)	12/23(52.17%)
	10.50 ± 0.29	9.10 ± 0.23	9.32 ± 0.28	9.47 ± 0.45
Infected group	67/94(71.28%)	46/85(54.12%)	38/53(71.70%)	29/36(80.56%)
	11.30 ± 0.28	10.48 ± 0.34	11.57 ± 0.58	12.29 ± 0.56
*χ2 value*	*1.164*	*4.323*	*6.26*	*4.077*
*P value*	*0.281*	*<0.05*	*<0.05*	*<0.05*
NEU(1.8–6.3*109/L)	*n* = 191	*n* = 164	*n* = 92	*n* = 59
Uninfected group	81/97(83.51%)	43/79(54.43%)	30/39(76.92%)	15/23(65.22%)
	8.63 ± 0.24	6.94 ± 0.20	7.12 ± 0.24	7.42 ± 0.41
Infected group	83/94(88.30%)	65/85(76.47%)	43/53(81.13%)	4/36(11.11%)
	9.12 ± 0.25	8.03 ± 0.31	9.16 ± 0.57	9.57 ± 0.52
*χ2 value*	*0.552*	*7.892*	*0.054*	*16.421*
*P value*	*0.458*	*<0.01*	*0.816*	*<0.01*
**NEU_P (40–75%)**	*n* = 191	*n* = 164	*n* = 92	*n* = 59
Uninfected group	89/97(91.75%)	46/79(58.23%)	24/39(61.54%)	15/23(65.22%)
	81.07 ± 0.48	75.78 ± 0.71	76.17 ± 0.71	77.68 ± 1.03
Infected group	81/94(86.17%)	50/85(58.82%)	37/53(69.81%)	28/36(77.78%)
	80.12 ± 0.48	75.88 ± 0.68	77.72 ± 0.89	77.85 ± 0.82
*χ2 value*	*1.003*	*0.007*	*0.368*	*0.575*
*P value*	*0.317*	*0.935*	*0.544*	*0.448*
**PCT(<0.5ng/ml)**	*n* = 188	*n* = 156	*n* = 84	*n* = 58
Uninfected group	31/95(32.63%)	32/75(42.67%)	20/36(55.56%)	12/23(52.17%)
	0.04 ± 0.00	0.05 ± 0.00	0.06 ± 0.00	0.06 ± 0.00
Infected group	50/93(53.76%)	51/81(62.96%)	29/48(60.42%)	18/35(51.43%)
	0.12 ± 0.05	0.12 ± 0.03	0.10 ± 0.02	0.08 ± 0.02
*χ2 value*	*7.718*	*5.654*	*0.05*	*0.045*
*P value*	*<0.01*	*<0.05*	*0.823*	*0.831*
**IL-6(≤ 7.0pg/ml)**	*n* = 116	*n* = 90	*n* = 60	*n* = 36
Uninfected group	24/59(40.68%)	1/43(2.33%)	2/24(8.33%)	1/11(9.10%)
	6.83 ± 0.50	2.96 ± 0.24	3.31 ± 0.46	4.09 ± 1.70
Infected group	26/57(45.61%)	10/47(21.28%)	5/36(13.89%)	6/25(24%)
	8.45 ± 0.94	6.50 ± 1.30	5.59 ± 1.45	6.98 ± 1.43
*χ2 value*	*0.122*	*5.855*	*0.061*	*0.341*
*P value*	*0.73*	*<0.05*	*0.806*	*0.559*

The data in Table 2 show the values of CRP, WBC count, NEU, NEU-P, PCT, and IL-6 between the uninfected and infected group on day1, day3, day5, and day7 after CC. In terms of CRP, there were no significant differences in day3, day5, and day7 after CC. However, the infected group showed a significant increase in day1 compared with the uninfected group (*P* < 0.05). Conversely, borderline significant differences were observed in WBC count on day3, day5, and day7 after CC (*P* < 0.05) except for day1. Moreover, the data also demonstrate significant differences in the NEU values on day3 and day7 after surgery between the uninfected and infected group(*P* < 0.01), whereas there were no significant differences on postoperative day1 and day5. For the NEU_P values, no significant differences were observed during the analysis. Furthermore, PCT values of the infection group indicated significant differences in day 1 (*P* < 0.01) and day 3 (*P* < 0.05) after CC compared with the uninfected group. Finally, it was found that IL-6 values differed significantly only on day3 (*P* < 0.05). Infection indices on other time points showed no significant differences.

**Fig. 5:**
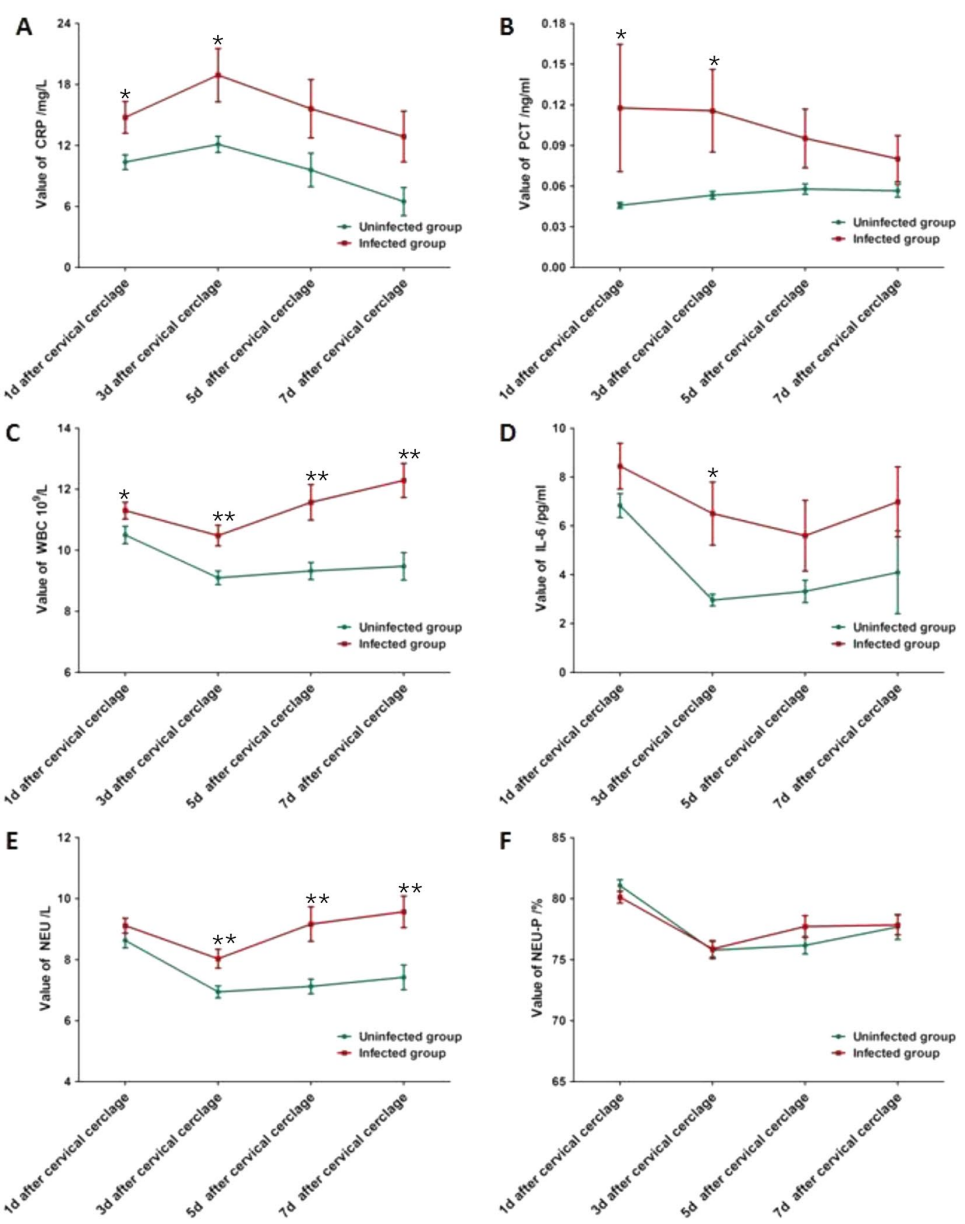
Mean values of CRP(**A**), PCT(**B**), WBC count(**C**), IL-6(**D**), NEU(**E**) and NEU-P(**F**) on d1, d3, d5 and d7 after CC in uninfected and infected groups(∗*P* < 0.05, ∗∗*P* < 0.01)

The changing trends of CRP, PCT, WBC count, IL-6, NEU, and NEU-P were measured and analyzed on day1, day3, day5, and day7 after CC(Fig. 5). In terms of CRP in both groups, the highest values were observed on d3, after which it steadily declined (A). Also, PCT values in the infected group showed a similar trend, which decreased gradually after day3. On the contrary, PCT values in the uninfected group presented the opposite trend and thoroughly remained normal (B). WBC count (C), NEU (E), and NEU-P (F) in the infected group reached their lowest values on day3 and then increased thereafter. In addition, IL-6(D) values in the infected group were elevated only on day1 and then returned to normal. Values of the uninfected group remained within the normal range after surgery.

**Fig. 6:**
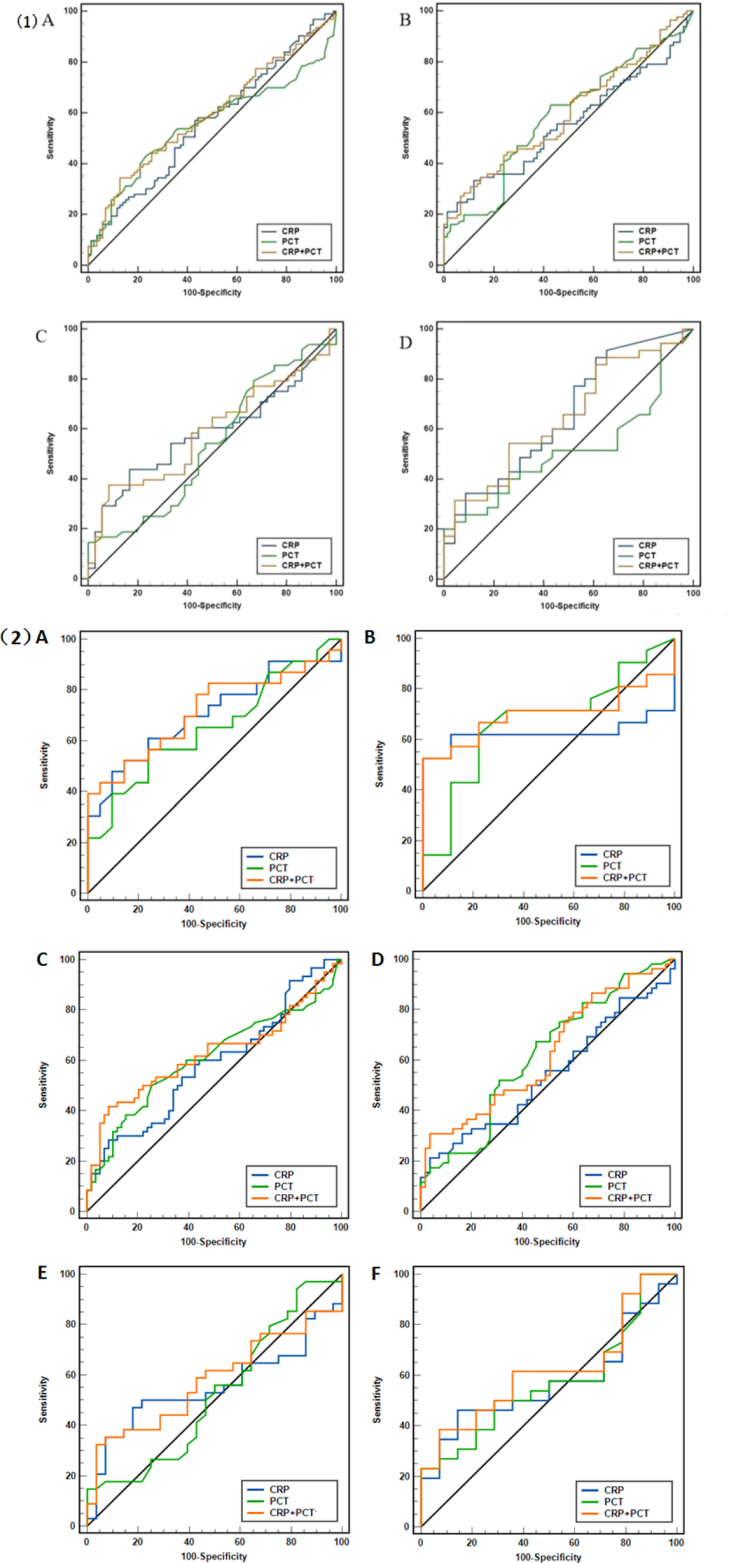
(**1**) ROC curves for CRP, PCT alone or in combination on d1(**A**), d3(**B**), d5(**C**) and d7(**D**) after CC. Figure 6 (**2**) ROC curves for CRP, PCT alone or in combination on d1(**A**), d3(**B**) after preventive CC and d1(**C**), d3(**D**), d5(**E**), d7(**F**) after non-preventive CC (therapeutic and emergency). The curves show how sensitivity (true positive fraction) varies with specificity (false-positive fraction) when the diagnostic cut-off limit is varied

ROC curves for each studied biomarker and combined biomarker after 1, 3, 5 and 7 days of CC are shown in Fig. 6(1). On day1 after CC(A), the AUCs under the ROC curves for CRP, PCT, and CRP-PCT are 0.56 (95% CI: 0.49–0.63), 0.55(95% CI:0.48–0.63) and 0.59(95% CI:0.52–0.66), respectively. Likewise, for d3(B), the AUC of CRP-PCT[0.60(95% CI:0.52–0.67)]was bigger than that of PCT[0.59 (95% CI:0.50–0.66)]or CRP[0.56(95% CI:0.47–0.63)] alone. In terms of d5(C), the AUCs under the ROC curves from largest to smallest were CRP-PCT[0.59 (95% CI:0.48–0.70)], CRP[0.58 (95% CI:0.47–0.69)] and PCT[0.53 (95% CI:0.42–0.64)]. However, on d7(D), the AUC for CRP accounts for the largest [0.66 (95% CI:0.52–0.78)], followed by CRP-PCT[0.65 (95% CI:0.51–0.77)] and PCT[0.52 (95% CI:0.38–0.65)].

Figure 6(2) presents subgroups results (preventive CC and non-preventive). On day1 of preventive CC (A), the AUCs under the ROC curves for CRP, PCT, and CRP-PCT were 0.71 (95% CI: 0.55–0.83), 0.65(95% CI: 0.49–0.79) and 0.72(95% CI: 0.56–0.84), respectively. And for day3 of preventive CC (B), the results were 0.62 (95% CI: 0.43–0.79), 0.67(95% CI: 0.48–0.83) and 0.70(95% CI: 0.50–0.85), respectively. The AUCs under the ROC curves for CRP, PCT, and CRP-PCT were all less than 0.70 on d1, d3, d5 and d7 in the non-preventive CC subgroup.

## Discussion

Infection poses a significant risk in the complications of transvaginal CC, particularly chorioamnionitis. Although the CC surgery itself does not significantly increase the risk of infection, cervical shortening can elevate the chances of ascending reproductive tract infections. It has been reported that the median infection rates of chorioamnionitis in patients undergoing preventive and non-preventive CC were 2% and 25%, respectively [17]. Therefore, close monitoring of infection indices after CC in a non-invasive manner is crucial. This study addresses the question of which day and which common infection indices should be focused on to detect potential infection after CC.

CI and genitourinary tract infections were identified as independent risk factors for premature birth (PTB) [18, 19]. In this retrospective study, pregnancy complicated with infection was found to be the most prevalent among risk factors for infections in patients with CI. Mycoplasma infection in the genital tract was the top risk factor, consistent with previous studies [20, 21], which reported an association between mycoplasma infection and adverse pregnancy outcomes, such as preterm labor and chorioamnionitis. Among patients with diabetes mellitus, 37 pregnant women in the study were diagnosed pre- or during gestation. This factor is a typical high-risk factor for infection during pregnancy. Population-based studies indicate that women with diabetes mellitus are at an increased risk for urogenital infection [22, 23].

WBC, along with NEU and NEU_P, are commonly used hematological indices to measure infection or inflammation, including in pregnant women. However, recent research has found that total WBC levels can significantly increase in pregnant women compared to the general population, with upper limits ranging from 13.8 to 19.6 × 10^9/L [24, 25]. Similarly, NEU levels can increase by 55% during pregnancy, according to a study of 24,318 healthy pregnant women [26]. In this study, WBC, NEU, and NEU_P were compared between the infected and uninfected groups on different days after CC. While WBC values in the infected group were significantly higher than in the uninfected group on days 3, 5, and 7, these values remained within the normal range, as mentioned in previous research. The same trend was observed for NEU. NEU_P values showed no significant differences between the two groups. Therefore, it is suggested that WBC, NEU, and NEU_P may have limited value in assessing infection in patients after CC surgery. This could be due to the increase in WBC levels during pregnancy, making it difficult to detect abnormal elevations that may occur after infection, as noted in a study by Chang J et al. [27].

CRP, produced by the liver [28], varies in healthy individuals with different demographic characteristics, including pregnancy [29]. Morimoto Y et al. [30]. reported that CRP levels increase as pregnancy progresses. In late pregnancy, Musilova I et al. [31]. stated that the utility of CRP is limited due to the late increase. In this study, since the appropriate surgical timing for CC focuses on the period before 28 weeks of gestation, the late increase in CRP is unlikely to impact its application in predicting infection after CC. The study found significant differences in CRP values on day 1 and day 3 after CC, suggesting that abnormal increases in CRP on these days may be indicative of infection. However, the low AUC values of ROC curves for CRP indicate low diagnostic accuracy. This finding is consistent with a study by Wiwanitkit V. [32], which concluded that maternal CRP estimation is not helpful in detecting chorioamnionitis. The results of Stepan M et al. [33]. also support the limited diagnostic value of CRP in intrauterine infection. However, this finding contradicts the study by Eun YJ et al. [34]. which reported that serum CRP has similar accuracy to amniotic fluid index in predicting intra-amniotic infection and/or inflammation among patients with CI or an asymptomatic short cervix. Additionally, Ji Wonk Park et al. [35]. also discovered that preoperative CRP levels could be considered a useful noninvasive marker, comparable to the concentration of amniotic fluid IL-6 in identifying pregnant women with CI at high risk of spontaneous preterm birth following rescue cerclage. This discrepancy may be due to differences in the characteristics of the included patients.

PCT, a biochemical marker for estimating inflammation and infection, has demonstrated high sensitivity and specificity in infection diagnosis [36]. Therefore, PCT is now generally accepted as a valuable reference index for guiding antibiotic prescribing in the general population. Due to these circumstances, PCT attracts the attention in the field of obstetrics [37, 38]. However, its application in obstetrics has yielded mixed results. One study found that PCT is less likely to be associated with maternal bacterial infection during pregnancy [39], consistent with the findings of Stranak Z et al. [40]. who suggested that PCT in patients suspected of chorioamnionitis is more likely to be released by the fetus rather than by maternal placental tissue. Conversely, Benita T et al. [41]. argue that PCT is a useful biomarker for severe bacterial infections in the field of Obstetrics and Gynecology. In this study, PCT values exhibited significant differences between the infected and uninfected groups on multiple days after CC. According to the research of Yun Hu et al. [42]. reference intervals for PCT among healthy Chinese pregnant women were 0.018–0.051 µg/L in the first and second trimesters. The results of PCT in this study provide meaningful reference values on postoperative day 1 and day 3, suggesting that infection should be considered when PCT values are elevated during the first three days after CC. However, similar to CRP, the AUC values of ROC curves for PCT and CRP-PCT were low, indicating limited diagnostic accuracy. The values of this study were lower than what was explored by Yan L et al. [43]. In their study, post-systemic immune-inflammation index(SII) and Δsystemic inflammation response index levels (SIRI) could help determine candidates before surgery and improve postoperative surveillance, which was in line with the conclusions of Selim G et al. [44]. They reported that SII presented a negative correlation with the duration of labor. Our data was obtained within 1 day postoperatively. With the early identification of suspected infected patients within 48 h, antibiotic treatment may be chosen to combat the suspected infections. Some patients showed a decrease in infection markers after antibiotic treatment, while severely infected patients displayed stable or increasing infection markers. What’s more, the varied pathogenic mechanism among preventive, therapeutic and emergency group could reduce diagnostic accuracy. Although these factors above resulted in relatively low AUC values for this research, CRP and PCT remain the most referenced infection markers among physicians, given their established significance in infected diseases, especially perioperative infections [45, 46]. The subgroup analyses did suggest that CRP and CRP-PCT obtained on day 1 may be referred to with moderate accuracy for patients undergoing preventive CC.

Some limitations and strengths of this study should be noted. One limitation is the single-center and retrospective study design, which may introduce bias related to patient characteristics. PSM was employed to mitigate this bias. Fortunately, the study center had a wide service coverage, with patients coming from nearly all regions in China, which helped alleviate the limitations associated with a single-center study. The second limitation is the exclusion of twin pregnancies, which was necessary due to potential physiological differences between mothers with single and twin pregnancies. Third, the newborns of patients with high infection values should better be recorded and analyzed as a primary endpoint to evaluate CC. This includes neonatal morbidity, neonatal infection markers and neonatal mortality. To date, we have not been able to access the corresponding information of premature babies taken to the NICU, as most of these babies are immediately transferred to pediatric hospitals for better treatment after birth. Future studies may focus on analyzing infections in pregnancies with a larger sample size, incorporating more infection indices and endpoints.

In conclusion, for women undergoing CC surgery, an increase in serum CRP and PCT on post-CC day 1 to day 3 may be an early sign of postoperative infection. Therefore, monitoring CRP and PCT during the first three days after surgery is essential, as there are significant differences between the infected and uninfected groups during this period, especially for patients undergoing preventive CC.

## Data Availability

The datasets used and/or analysed during the current study are available from the corresponding author on reasonable request.
